# Untangling the causes of geographical disparities in the clinical outcome of HTLV-1 infection: a critical perspective on the contribution of viral genetic diversity

**DOI:** 10.1128/jvi.00601-25

**Published:** 2025-07-07

**Authors:** Thomas Duchateau, Philippe V. Afonso, Chloé Journo

**Affiliations:** 1 Centre International de Recherche en Infectiologie, Retroviral Oncogenesis team, Inserm U1111 - Université Claude Bernard Lyon 1, CNRS, UMR5308, École Normale Supérieure de Lyon, Université Lyon, Lyon, France; 2 Unité Épidémiologie des Virus Oncogènes, Institut Pasteur, Université Paris Cité 555089, Paris, France; New York University Department of Microbiology, New York, New York, USA

**Keywords:** viral genetic diversity, viral oncogenesis, HTLV-1

## Abstract

Human T-cell leukemia/lymphoma virus type 1 (HTLV-1) is the etiological agent of two major diseases, adult T-cell leukemia/lymphoma (ATLL) and HTLV-1-associated myelopathy/tropical spastic paraparesis (HAM/TSP), as well as various other inflammatory manifestations. The prevalence of HTLV-1 infection varies greatly from region to region, and available epidemiological data support the notion that even among regions of similarly high prevalence of HTLV-1 infection, such as Japan, South America, or Central Australia, the incidence of each associated disease may vary drastically. Here, we critically review evidence supporting a high incidence of ATLL in Japan, in contrast to a high incidence of HAM/TSP in South America, and a low incidence of both diseases in Central Australia, where other HTLV-1 inflammatory diseases are common. We further aim to explore these singularities through the lens of HTLV-1 genetic diversity. Different genetic clades are geographically restricted, such as HTLV-1a-Jpn and HTLV-1a-TC in Japan, HTLV-1a-TC in South America, HTLV-1a-TC and HTLV-1b in Africa, and HTLV-1c in Australo-Melanesia. We analyze potential correlations between HTLV-1 genotypes and disease outcomes while also discussing the interaction with other genetic or environmental factors that may contribute to these differences (e.g., host genetic background, age of infection, routes of transmission, or environmental factors). This perspective raises important questions about the unique properties of the different HTLV-1 genotypes and how they may reshape our understanding of HTLV-1 pathophysiology on both molecular and epidemiological levels.

## INTRODUCTION

After its initial identification and isolation in 1980 in the United States (1), in a patient of African ancestry, and in 1981 in Japan (2), human T-cell leukemia/lymphoma virus type 1 (HTLV-1) was subsequently detected in several regions of the world, such as the Caribbeans (3, 4), South America (5), and Australo-Melanesia (6). Currently, HTLV-1 is considered to be present worldwide, but with an uneven distribution, with foci of endemicity including Southern Japan, Central and South America, the Caribbean, West and Central Africa, the Mashhad region of Iran, Romania, and Oceania.

HTLV-1 human-to-human transmission occurs either vertically, from mother to child, or horizontally, through sexual or parenteral transmission. In 5%–10% of infected individuals, infection by HTLV-1 leads to the development of either one of two severe outcomes: a hematological malignancy, named adult T-cell leukemia/lymphoma (ATLL) (7), that manifests decades after primary infection, or a neuroinflammatory and degenerative disease named HTLV-1-associated myelopathy/tropical spastic paraparesis (HAM/TSP) (4). Additional inflammatory manifestations have been associated with HTLV-1 infection, including HTLV-1-associated infective dermatitis (HAID) (8–10), uveitis (10, 11), Sjörgren syndrome (10, 12), as well as the respiratory disease bronchiectasis (10, 13). The World Health Organization now considers HTLV-1 infection to be a matter of concern in lower-middle-income countries with high prevalence, especially in Latin America and the Caribbean (14).

Over the past 30 years, numerous observations have noted significant geographical differences in the incidence of HTLV-1-associated diseases, with reports claiming the incidence rate of ATLL or HAM/TSP to vary by factors up to 20 times between populations. At first glance, it seems that ATLL incidence is higher in Japan, and HAM/TSP cases are more frequent in Brazil or the Caribbean; in Central Australia, other inflammatory diseases seem to be more prevalent (see “Difficulties in estimating and comparing incidences of HTLV-1-associated diseases,” below).

In this review, we aim to critically review the data allowing us to map the geographical disparities in the clinical outcome of HTLV-1 infection, by comparing the cumulative risks of HTLV-1-associated diseases at global and local geographical scales, and by stating the limitations of such comparisons. While multiple factors may contribute to these disparities (e.g., host genetic background, routes of infection, environment), we focus on one that has been neglected over the years, which is HTLV-1 genetic diversity. We review recent data on viral genotypes being an influencing factor of disease outcome and discuss the extent to which the genetic diversity of the virus may interact with other known parameters, such as transmission routes and host genetics. In a nutshell, this study aims to disentangle the bases of the geographical singularities (in behavior, host genetics, and environment) in the clinical outcome of HTLV-1 infection.

## GEOGRAPHICAL DISPARITIES IN THE CLINICAL OUTCOME OF HTLV-1 INFECTION: A CRITICAL COMPARISON OF CUMULATIVE RISKS OF HTLV-1-ASSOCIATED DISEASES

Incidence is the rate of new cases over a specified period for a given population, either the general population or specifically the population at risk (i.e., HTLV-positive). The lifetime risk is the probability, over the lifespan, of developing the disease when infected. Of note, as the median survival of ATLL and HAM/TSP patients is very different, the prevalence of the disease does not directly reflect its incidence. As an example, in Japan, the incidence of ATLL is estimated to be 1,000 new cases per year, while HAM/TSP incidence is estimated to be 40 new cases per year overall (Yamano, unpublished data). As the median survival of ATLL patients is lower than 1 year, while that of HAM/TSP patients is higher than 16 years, it is estimated that at any given time, there are approximately 1,000 ATLL and 640 HAM/TSP patients in Japan.

### Difficulties in calculating the prevalence of HTLV-1 infection

First, the estimation of prevalence depends on the method used. Historically, data were drawn directly from serological methods, which may have overestimated HTLV-1 prevalence. For instance, ELISA tests are known to significantly cross-react with African parasites (15). Namely, false-positive HTLV-1 tests have been associated with cross-reactivity with *Plasmodium falciparum* in Central Africa, with a profile named HTLV-1 Gag Intermediate Profile (HGIP) (16). Moreover, several indeterminate western blot profiles have been observed in South Cameroon but were found negative by PCR. Nowadays, most studies use a two-step prevalence estimation, with a first-line serological screening followed by molecular validation by PCR. However, the fact that a subset of indeterminates might in fact represent *bona fide* exposure to an infection with HTLV-1 has been documented. In these cases, such a status is linked to a very low proviral load (PVL) (17, 18), hindering the detection of HTLV-1 provirus even with PCR analysis (19), or to the presence of incomplete or truncated proviruses. Moreover, in some cases, longitudinal studies of individuals with indeterminate profiles show eventual seroconversion, suggesting that these individuals were likely recently infected and in the early stages of developing a humoral immune response. The indeterminate profile thus appears to be a marker of recent infection (20). Given these observations, excluding PCR-negative indeterminate profiles might lead to underestimation of HTLV prevalence, and, in turn, impact the estimation of disease incidence in endemic areas.

Second, to estimate the incidence of HTLV-1 diseases, it is first necessary to estimate the size of the population at risk of developing HTLV-1 diseases, that is, the size of the infected population. HTLV-1 prevalence is usually estimated from sample populations with readily available serological data, such as pregnant women or blood donors. The choice of the sample population may have an impact on the calculated incidence, since the considered population segment may have a specific mean age, sex ratio, sociological and educational status, etc.

When considering pregnant women, estimated HTLV-1 prevalence varies up to 20 times geographically, from 0.045% in Europe (21) to 0.84% in Brazil (22). In endemic countries, HTLV-1 prevalence in pregnant women may also vary at the regional scale, for instance, in Brazil, with prevalence ranging from 0.11% in Mato Grosso do Sul, up to 1.05% in Bahia (23). Geographical disparities have also been reported in African endemic countries such as Gabon (from 2.1% up to 6.8%) (15, 24) and Guinea-Bissau (from 2.2% to 3.3%) (15). However, when extrapolating comparisons of HTLV-1 prevalence from data on pregnant women from distinct continents, it is important to highlight that the average age of pregnant women may vary significantly from continent to continent. Since HTLV-1 prevalence is known to increase with age, this may bias the actual comparison.

Regarding blood donors in Japan, the total estimated prevalence based on first-time detected blood donors is approximately 1% (25), but it can reach 30% in Southern regions (26, 27). The prevalence in Western and Central Africa is among the highest worldwide, exhibiting significant variations between and within countries (28). The prevalence can reach 5% in the urban adult population in Gabon (29), with recent descriptions ranging from 2% to 12% in rural or urban populations, respectively (30, 31). But it should be noted that in Central Africa, the blood donor population is often younger and more male-biased when compared to other areas of the world.

Rare studies have estimated the viral prevalence using randomly sampled populations, and this approach confirmed stark geographical disparities. For instance, the Health Ministry of Brazil reported a high prevalence of HTLV-1 in state capital cities, up to 0.5% in Acre or Bahia. Again, high local disparities have been documented (32), since the prevalence in isolated populations with Afro-American origins has been shown to reach 5% (33). In Jamaica, the prevalence is estimated to be around 6% (34). HTLV-1 also circulates actively in the Vanuatu archipelago, where approximately 1% of adult aborigines have tested positive (35–37). The highest prevalence of HTLV-1 has been reported in Indigenous populations of Central Australia, with initial estimates of 7%–14% among Aboriginal males (38). Of note, Central Australia is the only region in the world where prevalence among men is higher than among women, reaching up to 30%–64% in the Alice Springs region (39, 40).

It is important to note that, although working in a given region, biases may arise from differences in sampling regarding ethnic origins. In Maripasoula, French Guyana, Noir-Marron populations are at a higher risk of infection than Creole populations, with a difference of a factor of 5. Similarly, in rural areas of Cameroon, Pygmy populations are 2.6 times more at risk of infection than Bantu populations (41).

Despite these limitations, these observations underline the geographical disparities, both globally and locally, in HTLV-1 prevalence, which in turn define the size of the population at risk of developing HTLV-1 diseases.

### Difficulties in estimating and comparing incidences of HTLV-1-associated diseases

Once the denominator is determined, determining the numerator, that is, the number of actual cases of disease among the population of interest, is a second challenge.

A first limitation in the accuracy of the diagnosis and notification of HAM/TSP vs. ATLL cases is that it greatly depends on the medical specialty to which HTLV-infected patients are referred to, which, in turn, may vary from country to country, from hematologists or oncologists (who might be most likely to diagnose ATLL) or neurologists (who might be most likely to diagnose HAM/TSP). In addition, the diagnostic criteria are not the same between regions. For instance, the criteria for HAM/TSP diagnosis are slightly different in Japan (Osame’s criteria) and Brazil (IPEC-2). Moreover, since Brazilian cohorts are mostly followed by neurologists, people living with HTLV-1 and developing prodromal symptoms may be qualified as “early” or “incident” HAM/TSP patients, while they would have initially been disregarded in other areas like the Caribbean.

Second, missed diagnoses may be frequent due to the lack of awareness toward HTLV-1-induced diseases or to the absence of systematic HTLV screening, leading to the number of cases being underestimated. Moreover, some ATLL may be assigned to closely related diseases, such as Sézary syndrome. Finally, the published incidence numbers depend on the method of data collection: in Japan, a proactive review of the national registries led to the identification of 1,000 cases per year (13). No such thorough review of registries has been performed in Brazil, which may explain, at least in part, why there have been only 12 new ATLL cases reported annually (14).

Of note, the most relevant method to calculate the incidence of a disease is to follow up a group of people at risk in a prospective longitudinal cohort study, rather than to perform a cross-sectional study. However, the value for incidence will depend on the structuration of the cohort. For instance, a recent prospective study in French Guyana enrolled women at pregnancy, with a median age at enrollment of 28, which might make it difficult to compare to other studies performed in Japan, for instance, where enrolled individuals were aged 60+ (42). Thus, the available studies estimating incidences in specific geographical areas or restricted cohorts are rarely fully comparable, due to the accumulation of confounding factors, including mean age, sex ratio, prevalence, socio-economic status, etc. Age of disease onset is another crucial parameter to consider for an accurate geographical comparison in incidence rates, since it seems to vary among distinct populations living with HTLV-1. Direct comparison in age- and sex-matched cohorts of Japanese and Jamaican patients, for instance, showed that the median age of ATLL patients differed significantly (43), the median age of ATLL being approximately 60–70 in Japan, compared to 40–50 in South America (44, 45), and this median age is only 42 in French Guiana (42) and 37 in South Africa (46). In the populations with early onset of ATLL, prognosis is also poorer, as illustrated in patients of Noir Marron ethnicity in French Guiana, compared to the Japanese cohorts (47).

Taken together, the main limitations to accurate estimates and comparisons are the availability of medical structures, the availability of molecular tools for viral screening (especially confirmatory methods), and the presence or absence of local specialists. Such a critical comparison between studies on ATLL incidence has been published recently (42), and Table 1 summarizes HAM/TSP incidence rates and cohort characteristics from available estimation studies and cohort studies. Despite all the presented limitations, these comparisons suggest geographical variation in the incidence rates of HTLV-1-associated diseases.

**TABLE 1:** HAM/TSP incidence rates estimated from retrospective surveys and prospective cohort studies^*g*^

	Kaplan et al. (48)	Murphy et al. (49)	Romanelli et al. (50)	Tanajura et al. (51)	Marcusso et al. (52)	Rosadas et al. (53)
Number of incident HAM/TSP cases	397, including 170 for the 1982–1988 period^*a*^	0	7	5^*d*^	12	4
HAM/TSP incidence rate (per 1,000 PY)	0.03	0	5.3	NA^*e*^	11.7	1.98
Total Person-Years (PY)	NA	272	1,315	NA	1,020	2,022
Median (or mean) follow-up	7 years^*a*^	2 years	7 years	3 years	5 years	6.8 years
Population	Patients from medical institutions (for HAM/TSP reports), blood donors (for prevalence estimates)^*b*^	Blood donors	Blood donors	Blood donors and other sources^*f*^	Individuals living with HTLV-1	Individuals living with HTLV-1
Median (or mean) age at enrollment	NA	40–49^*c*^	45.6	46.5	52	46.8
Median (or mean) age of incident HAM/TSP patients	45	NR	NA	NA	64	57
% women in the original cohort	NA	71.3	58	59	74	76
% women in incident HAM/TSP patients	71%^*a*^	NR	NA	NA	66.7	100
Cohort study	National survey of HAM/TSP	REDS cohort	GIPH cohort	HTLV-1-infected individuals	HTLV outpatient clinic of the Institute of Infectious Diseases Emilio Ribas (IIER)	National Centre for Human Retrovirology (NCHR)
Location	Eight prefectures from Okinawa and Kyushu Islands, Japan	USA	Brazil (Minas Gerais)	Brazil (Bahia)	Brazil (São Paulo)	UK

^*a*^ This study is not a prospective study but a retrospective survey-based study that included 589 reported HAM/TSP cases from 810 medical institutions in Japan, among which 397 cases from the eight prefectures were further discussed in the report, and 170 incident cases for the period of interest (1982–1988).

^*b*^ Seroprevalence data obtained on blood donors from the eight prefectures were used to estimate the incidence of HAM/TSP among people living with HTLV-1.

^*c*^ The exact median age is not provided, but the stratification of the cohort according to age is.

^*d*^ 78 additional individuals developed probable HAM/TSP.

^*e*^ All five individuals with HAM/TSP incidence entered the cohort with probable HAM/TSP, and the exact incidence rate of HAM/TSP is not calculated in this report.

^*f*^ Other sources from which individuals were referred to the cohort include television and radio advertisements, patient associations, community associations, the neurology clinic, and family members.

^*g*^ Characteristics of reported cases, and/or of the cohort used for follow-up are summarized. NA, not available. NR, not relevant.

Of note, despite the high prevalence of HTLV-1 infection among remote populations in Australia, ATLL and HAM/TSP manifestations are relatively rare, suggesting a low risk of these HTLV-1-associated diseases in these populations living with HTLV-1 (40). In endemic Central Australia, there are only 20,000 individuals who consider themselves indigenous. The prevalence reaches 30%–40% in the population, and about half of the population is 30 years and older. Therefore, the expected number of reported ATLL cases (for an average incidence rate of 2–4 cases per 1,000 persons per year) should be about 10–20 cases. There have, however, been no such frequent reports. This also holds true in some countries of Southern, Western, and Central Africa, although this might in part be related to the challenges in diagnosing and reporting these diseases in these areas (15).

Other HTLV-1-associated diseases exhibit a heterogeneous geographical distribution among people living with HTLV-1. For instance, HAID is reported most frequently in South America and the Caribbean (8, 9, 54), and very rarely in Japan, supporting the fact that HAID might primarily be concentrated in specific geographical areas (55–57). Of note, HAID patients are more at risk of developing HAM/TSP, purportedly because these are both inflammatory diseases and might share similar pathogenic steps (58). Intriguingly, bronchiectasis and inflammatory diseases associated with HTLV-1 infection are over-represented in Australia, but HAM/TSP is rarely reported (39, 59). Importantly, in Australia, HTLV-1c mainly circulates in socially disadvantaged Indigenous people; environmental factors and/or coinfection (with enteric pathogens) may favor the development of peculiar HTLV-1-associated inflammatory diseases, such as bronchiectasis, lower respiratory tract infections, and asthma (40). Interestingly, one of the first analyses of the immune profile of HTLV-1c-infected subjects revealed a pro-inflammatory context induced by this genotype, in particular in patients with pulmonary manifestations, with immune activation and pro-inflammatory cytokine production, as well as possibly lung-homing lymphocytes (communication at the HTLV conference, London, 2024). Altogether, despite the low prevalence of ATLL and HAM/TSP in Australian Indigenous people, HTLV-1c infection remains a major cause of mortality in these resource-poor areas.

Taken together, these observations underline geographical singularities in the incidence of HTLV-1-associated diseases. Interestingly, phylogenetic analyses have demonstrated the circulation of specific HTLV-1 genotypes in different geographical areas, raising the hypothesis of genotype-specific risks of HTLV-1-associated diseases.

## HTLV-1 GENETIC DIVERSITY COULD CONTRIBUTE TO DIFFERENCES IN PATHOGENESIS

### The analysis of HTLV-1 genetic diversity indicates that most HTLV-1 genotypes and subtypes are geographically restricted

**Fig 1:**
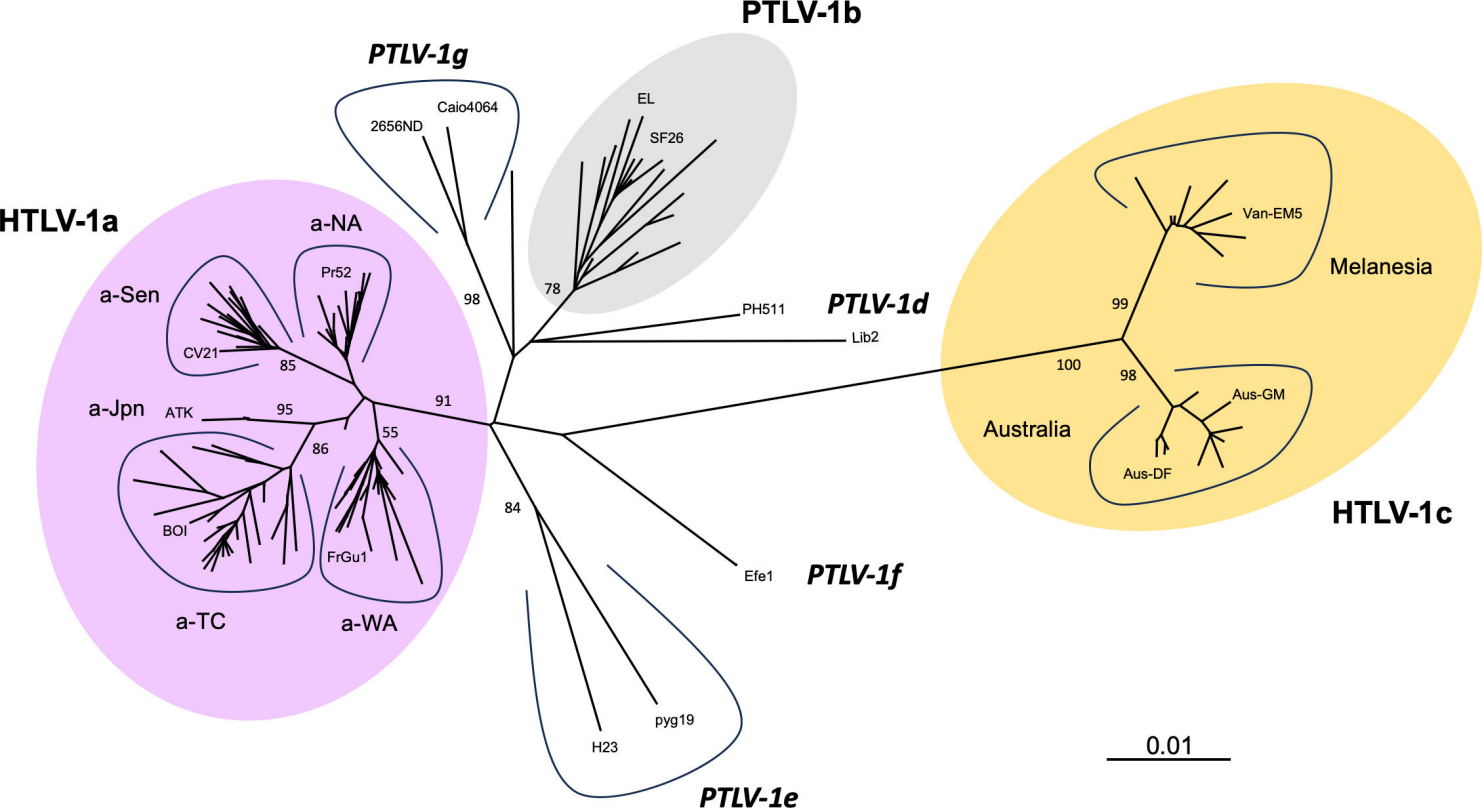
HTLV-1/PTLV-1 phylogeny. Phylogenetic alignment is based on complete LTR sequences (774-nt long) from 178 strains. The unrooted phylogenetic tree was generated with the neighbor-joining method using the GTR model (gamma = 0.4953). Branch lengths are drawn to scale, with the bar indicating 0.01 nucleotide replacement per site. PTVL-1 refers to genotypes for which at least one strain has been identified in non-human primates. HTLV-1a and HTLV-1c strains have been exclusively detected in humans so far, and their zoonotic origin is therefore currently unknown. HTLV-1c is currently the most divergent subtype of HTLV-1 with important intra-group divergence. Numbers on each node indicate the percentage of bootstrap samples (of 1,000 replicates).

The historical prototypic HTLV-1_ATK_ strain was isolated from a patient of Asian descent in 1981 in Japan (2). Since then, many other HTLV-1 partial or full-length genomes have been sequenced, allowing the identification of genotypes and subtypes (60). HTLV-1 phylogeny is based on alignment of the long terminal repeats (LTR) sequences, but has also been validated based on viral open reading frames (ORFs) such as *env* and *tax*, or concatenation of ORFs *gag pol env tax* (Fig. 1).

The HTLV-1_ATK_ strain belongs to the HTLV-1a genotype, which is qualified as “cosmopolitan” as it is present on every continent (60). However, the HTLV-1a genotype encompasses several major clades which are restricted to specific geographical areas, such as the Japanese (a-Jpn), the Western African (a-WA), and the Senegal clades (a-Sen). Some minor clades may have originated from recombination, such as the G-Rec and North African (a-NA) subgroups (60). All other HTLV-1a strains belong to the so-called “transcontinental” clade (a-TC), which is detected in multiple areas of the world.

Until very recently, most if not all molecular and epidemiological studies on HTLV-1 were performed on HTLV-1a strains, neglecting the diversity of HTLV-1 strains outside this clade. The greatest diversity of HTLV-1 genotypes is described in Africa. The most prevalent African genotype is HTLV-1b, which is present in Central Africa. The virus can be acquired through the same infectious route as HTLV-1a (see “Local behavior: transmission routes and age of infection,” below) (61), and can also be acquired upon severe bites by infected simian hosts (62). HTLV-1d, although less frequent, seems to have the same characteristics as HTLV-1b, that is, of human and zoonotic origin (63). Other minor African clades include HTLV-1e-g. Although a few sporadic cases have been reported for these clades, interhuman transmission remains to be demonstrated formally.

In addition to these African genotypes, another non-HTLV-1a clade has been documented: HTLV-1c, which is the most divergent HTLV-1 clade and which is restricted to Oceania. Two HTLV-1c subgroups have been identified: the Australian and the Melanesian clades (Fig. 1) (60).

The singular geographic distribution of HTLV-1 genotypes, combined with the geographical disparities in the clinical outcome of HTLV-1 infection described above, raises the hypothesis of genotype-specific risks of HTLV-1-associated diseases. However, it is difficult to disentangle virus-intrinsic properties from epidemiological, environmental, or host genetic factors that might be at play in viral pathogenesis. To overcome this challenge, interesting data have been obtained by studying comparable populations infected with different viral strains.

### Direct evidence of specific pathogenic properties of HTLV-1a genotypes: comparing HTLV-1a-Jpn and a-TC

In the Kagoshima region in Japan, both HTLV-1a-Jpn and a-TC strains circulate (64). Such a context is the most appropriate to interrogate the influence of the viral genotype by minimizing (although not abolishing) the biases due to host genetics, socioeconomic environments, and lifestyles. In this region, HTLV-1a-TC has demonstrated a more frequent association with HAM/TSP compared to HTLV-1a-Jpn, in a cohort controlled for the frequency of the HLA-A*02 allele. By contrast, the frequency of HTLV-1a-Jpn is comparable among individuals with HAM/TSP, ATLL, and healthy carriers (64–66). These results support the notion that viral-intrinsic properties might contribute to differing disease outcomes. This notion is also compatible with observations made at a larger geographical scale, although these are more difficult to control (as discussed in the following sections). As stated above, in Japan, where HTLV-1a-Jpn is predominant, the risk of HAM/TSP is much lower than in Jamaica and Colombia, for instance, two highly 1a-TC endemic areas (48, 67–69). Although not formally demonstrated, these observations are compatible with the hypothesis of a HTLV-1a-Jpn vs*.* 1a-TC-specific impact on disease outcome.

This hypothesis is further supported by molecular evidence. In the late 1990s, mutations in the viral sequence encoding the regulatory Tax protein have been proposed to contribute to differences in the risk of HAM/TSP associated with HTLV-1a-TC or 1a-Jpn subtypes (70). Although initial observations were refuted (71), subsequent discussions have supported the notion that Tax1a-TC is more strongly associated with HAM/TSP due to the presence of a specific pool of mutations in the coding sequence (64–66). A molecular comparison of Tax1a-TC- and Tax1a-Jpn-expressing cells revealed distinct transcriptional signatures, with a higher induction by Tax1a-TC, both *in vitro* and *in vivo*, of the gene encoding CXCL10, a factor known to play a crucial role in HAM/TSP (72). Experiments on Tax-inducible Jurkat T cells and PBMCs from patients indicated no difference in the capacity of NF-κB promoter activation between these subtypes, despite the identified mutations in Tax sequences being in proximity to p100 and NF-κB interaction sites (72). Nevertheless, Tax1a-TC demonstrated a greater affinity for both κB1 and κB2 transcription sites using ChIP assays, suggesting a specific transcriptional signature of both Tax variants (72).

While Tax is central in the pathogenesis of HTLV-1, many other viral regulatory sequences or open reading frames could be implicated in the molecular specificities of HTLV1a-TC vs. 1a-Jpn. For instance, the LTR relies on Tax-responsive elements (TRE) to promote provirus expression, and polymorphisms on these regulatory sites would result in varying rates of viral transcription. In 2021, Gomes et al. demonstrated that polymorphisms in Tax-responsive elements affect the PVL in HAM/TSP patients, despite no discernible impact on established disease progression (73). Furthermore, LTR polymorphisms are frequent in the HTLV-1a-TC subtype strains, correlating with the PVL and the risk of HAM/TSP (73). Increased viral gene expression may also enhance the recognition of infected cells through MHC presentation, resulting in a robust cytotoxic T lymphocyte (CTL) response, which, together with a high PVL, is a well-established marker of the disease.

Differences among viral genotypes have also been reported for HTLV-1 bZip Factor (HBZ), another viral regulatory protein. Indeed, it has been shown that HBZ1a-TC is more expressed than HBZ1a-Jpn in HAM/TSP patients (74). However, the functional implications of such an observation remain hypothetical.

**Fig 2:**
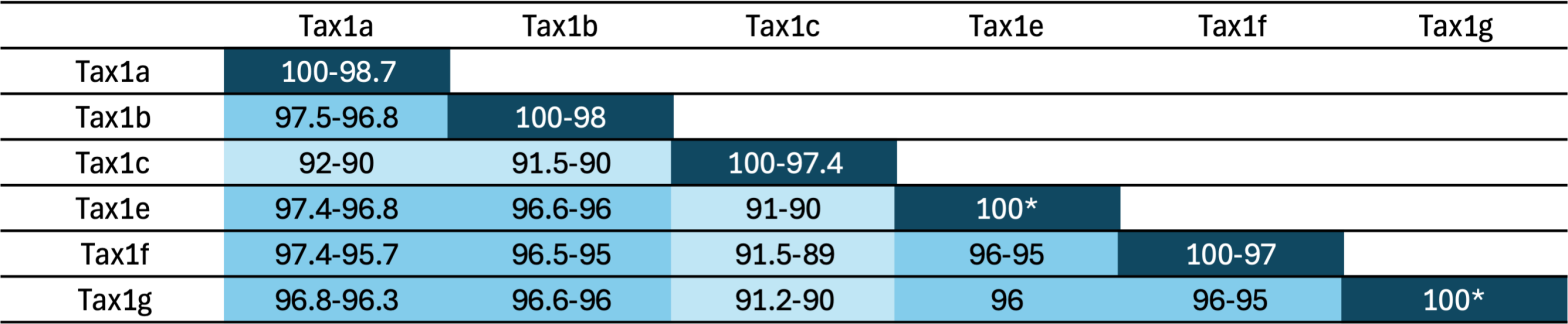
Comparison of Tax1 sequences among HTLV-1 genotypes. Publicly available Tax1 sequences were aligned, and similarity scores are reported in this table. For genotypes for which multiple Tax1 sequences are available, intra-genotype variation is reported. *One single sequence available. Tax-1d is not presented as sequences are not available yet.

These observations suggest that subtle variations in the viral genome between closely related HTLV-1a strains can quantitatively and qualitatively influence the expression of viral genes, as well as the functions of viral proteins, possibly impacting infection outcomes for both ATLL and HAM/TSP. We postulate that molecular differences along even more distantly related variants of HTLV-1, including HTLV-1b, c, d (see Fig. 2 for genetic distances among Tax sequences from distinct genotypes), could also contribute to variation in pathogenesis. While this has not been evaluated comprehensively so far, interest in HTLV-1c in Australia has significantly increased over the past few years, and recent data have shed light on the specificities of this clade.

### Divergence of HTLV-1c and other non-HTLV-1a viral genes and implications in viral pathogenesis

Geneveffa Franchini’s and Christophe Nicot’s laboratories have long been interested in the encoding of small auxiliary proteins by HTLV-1 and have found that these often-forgotten proteins have central roles in viral persistence. For instance, it has been recently shown that an equilibrium between p12 and p8 proteins is essential for viral propagation during primary infection and persistence of the virus over the years (75, 76). In addition, in 2002, Martins et al. suggested that a mutation on the auxiliary protein p12 could be associated with an increased risk of developing HAM/TSP (77). Intriguingly, HTLV-1c strains, although capable of transmission and persistence, are mutated on the p12 ORF. This mutation on the start codon of p12 leads to the absence of translation of the viral protein (78). Moreover, in Australia, analysis of strains of HTLV-1c circulating in Indigenous populations has shown that many people living with HTLV-1 harbor a truncated provirus (78). It is currently unclear how mutations on the p12 start codon or truncations in the provirus can affect viral persistence. Similarly, we have recently performed *in silico* analyses and found that ORFs encoding p30 and p12 may be defective in many HTLV-1 strains (79), which could impact proviral load, clonal expansion, and pathogenesis. Other viral proteins are also modified between subtypes, especially p13 encoded in ORF-II and involved in pathogenesis (80), but the consequence of such modifications in viral pathogenesis is still under investigation.

### Could animal models help dissect the specific properties of a given HTLV-1 genotype?

The observations summarized above support the notion that viral genotypes may display specific properties that could directly contribute to pathogenesis. Comparing the properties of distinct HTLV-1 genotypes in an animal model could represent an interesting strategy; however, in the context of HTLV-1 research, this is not trivial and has not been done yet.

When investigating the oncogenic and inflammatory potential of individual viral products, transgenic animal models can be used. Transgenic mice have primarily been used to demonstrate the oncogenic activity of Tax (81) and HBZ (82) from the HTLV-1a genotype. More uniquely, transgenic *Drosophila melanogaster* models have also been harnessed to assess the oncogenic power of HTLV-1a Tax and HBZ, expressed alone or in combination (83, 84). Interestingly, in the fly model, comparisons between HTLV-1 and HTLV-2 Tax proteins have been performed (83): this model could also be employed to compare the functional properties of Tax variants from distinct HTLV-1 genotypes. This model has its limitations; however, since only one or two viral factors are expressed, in the absence of the full virus, and is far from recapitulating the complex host/pathogen interactions that are at play in infected individuals and that lead to pathogenesis.

Experimental HTLV-1 infections in the rabbit models have been extensively used, in particular by Michael Lairmore’s and Patrick Green’s groups. Rabbits serve as a good model for the initial steps of HTLV-1 infection, from establishment to persistence, although they do not develop HTLV-1-associated diseases (for a review, see reference 85). Taking advantage of stable producer cell lines, generated using HTLV molecular clones, to inoculate the animals, the rabbit model has been used to compare HTLV-1 and HTLV-2 (86), and also to assess the contribution of specific viral products (87), or specific domains of viral proteins or features of viral transcripts (88, 89), in an infectious setting. Stable producer cell lines producing wild-type or mutant HTLV-1 or HTLV-2 have also been used to experimentally infect humanized mice, which develop symptoms, or macaques (for a review, see reference 84). Should stable producer cell lines producing HTLV-1b, -1c, or other genotypes be available, experimental infections with distinct subtypes could be set up.

Nonetheless, other factors influence natural HTLV-1 infection, both in humans and non-human primates, such as the age of infection, the transmission routes, and the host genetics, all of which are poorly recapitulated in experimental infections. In the following section, the contribution of these other factors in human infection, as well as their possible interaction with the viral genotype, will be discussed.

## OTHER FACTORS THAT COULD CONTRIBUTE TO DIFFERENTIAL DISEASE OUTCOME, OR THAT COULD BIAS ANALYSIS

### Local behavior: transmission routes and age of infection

A major confounding factor between viral genotype and disease outcome could lie in local differences in the preferential route of viral transmission and age of viral acquisition. Demonstration of the influence of transmission route and age of infection on the development of ATLL and HAM/TSP has been a major output of HTLV-1 research during the last 40 years, raising the hypothesis that it could partially explain the geographical variation in HTLV-1-associated diseases. Age and mode of transmission could affect the stage of immunity at primo-infection, as well as the quantity and quality of viral inoculum, which is thought to be reduced in horizontal transmission compared to breastfeeding. Here, we question the interplay of the viral genotype with these epidemiological parameters.

#### Oral infection in infants and blood or sexual infection in adolescents and adults are associated with increased risk of developing ATLL and HAM/TSP, respectively

Vertical transmission primarily occurs through prolonged breastfeeding (longer than 6 months) (90) and is now accepted to be associated with a substantial risk of ATLL development (91, 92). Increased susceptibility to ATLL development following vertical transmission could be linked to stochasticity in the clonal expansion of infected cells, limited in early infected compared to adult seroconverts (93). Conversely, infection acquired sexually or through blood contact during adolescence or adulthood is more likely associated with the development of HAM/TSP (94). Indeed, while a high number of lifetime sexual partners is not significantly associated with the risk of ATLL development, it is a significant risk factor for HAM/TSP development (68). In addition, the age of first sexual intercourse was reported to be significantly lower in HAM/TSP patients compared to asymptomatic carriers, and the interval between the first sexual intercourse and the development of HAM/TSP correlated with the age of first sexual intercourse (68). Thus, given that both the age and the route of infection influence the risk of developing HTLV-1-associated diseases, geographical variations in these parameters could contribute to the geographical disparities in HTLV-1-related diseases.

#### Could interaction between varying transmission routes and viral genotypes contribute to the geographical disparities in HTLV-1 infection outcome?

Very little is known about the transmission routes of HTLV-1 variants, and most specifically the relative contribution of vertical or horizontal transmission. Comparing the relative contribution of each transmission route among distinct endemic areas or viral genotypes is challenging. Horizontal transmission appears to be dominant in HTLV-1a-TC-endemic areas, especially in South America (95, 96), suggesting that viral genotype and horizontal transmission could synergistically predispose these populations to HAM/TSP. This is, however, not observed in every population: for instance, in French Guiana, where HTLV-1a-TC circulates, pregnant women of the Noir Marron population have a very high prevalence of HTLV-1 infection (42), leading to a very high risk of vertical transmission and a very high incidence of ATLL (2 cases per 1,000 HTLV-1 carriers in a follow-up of 16.7 years with a median age at diagnosis of 47, against 0.5 cases in equivalent Japanese cohorts in a follow-up of 13 years with a median age at diagnosis of 65–70; it should be noted that the age structuring of both cohorts differed significantly, though) (97).

In Central Africa, where genotypes HTLV-1b-g are most prevalent, several studies have postulated that transfusion could be a significant mode of transmission among hospitalized individuals, due to the absence of systematic blood screening (15, 98, 99). However, the contribution of parenteral transmission in Africa is challenged by other studies. In Gabon, for instance, the acquisition of HTLV-1 is not always significantly associated with the transfusion history of individuals, even in recent literature (31, 61, 100). Conflicting results are also observed in other countries, such as Guinea-Bissau, where HTLV-1 infection appears independent of blood transfusion (101). Conflicting observations regarding the contribution of parenteral transmission of HTLV-1 in Africa could in fact be partially explained by the specific viral genotype circulating in these areas, which is not systematically documented. It is important to note that in Europe, where screening of organ donors and leukoreduction in blood donations is mandatory, the transmission of HTLV-1 after organ transplant or blood transfusion is virtually nonexistent, which is not the case in Africa, where such policies are not applied. In Australia, no association between the risk of HTLV-1 acquisition and the history of blood transfusion has been detected, but very few studies have investigated this aspect in this endemic area (102). The contribution of parenteral transmission to the circulation of HTLV-1 could therefore greatly vary geographically, either due to different characteristics of the viral strains or to different socioeconomic contexts.

Transmission through breastfeeding has recently been reported as similar between cohorts in Japan and Australia, with rates around 3% (103, 104). Since Japan has the highest incidence rates of ATLL among all endemic areas, while Australia has one of the lowest rates (40, 45), this suggests that the oral mode of transmission itself might not be the only factor influencing the variable risk of ATLL development between these regions.

Noteworthily, other non-conventional transmission routes have been documented in specific areas of the world, linked to specific cultural practices. In Guinea-Bissau, cultural ornamental scarification rites have been demonstrated to serve as a blood-contact route for HTLV-1 transmission (105). Flagellation has also been suggested as a possible route of HTLV-1 transmission through blood (106). Similarly, among Indigenous populations in Australia, ritual scarification during the passage to adulthood in young men might contribute more significantly to the circulation of HTLV-1c than initially expected (15, 103). Consistently, in these populations, HTLV-1 prevalence in men sharply increases between 4.1% in the 5–14 age group and 37.3% in the 15–24 age group, which represents a more significant increase than in women (103). In contrast to any other endemic region worldwide (104), Australian Indigenous men are much more likely to be infected by HTLV-1 than women. However, whether these non-conventional transmission routes can influence the establishment of infection, HTLV-1 chronicity, and the onset of diseases is currently unknown. Interestingly, in Australia specifically, the most prevalent comorbidity of HTLV-1 infection is inflammatory diseases of the pulmonary tract, which are overrepresented in seropositive cohorts and affect up to 17% of individuals living with HTLV-1 (13, 40). These inflammatory diseases, and especially bronchiectasis, are associated with a high PVL (up to 100-fold higher than in asymptomatic carriers) (107), as described for HAM/TSP patients (108). However, attributing these clinical differences to the transmission route or the HTLV-1c genotype specifically, in the context of a specific socioeconomic environment, is challenging.

The relative contribution of transmission routes or viral genotype might be best disentangled in specific contexts where public health policies have been implemented that modify the circulation of the virus, while not affecting the relative frequencies of circulating viral genotypes. For instance, in Japan, the relative contribution of vertical transmission has declined recently (109, 110). In the 1980s, childhood infection through breastfeeding was considered to contribute significantly to HTLV-1 circulation in Japan, with over 80% of seropositive individuals having a seropositive mother (111). Implementation of preventive measures in the late 1980s and 1990s, in the form of recommendations that positive pregnant women refrain from breastfeeding, has led to a significant drop in the risk of vertical acquisition of the infection, from 15%–20% to 2%–3% (112, 113). On the other hand, horizontal transmission in Japan still represents almost 3,000 new cases per year in young adults (109). Despite these important shifts in the relative contribution of transmission routes, no effect on the number of ATLL cases declared per year has been observed yet (114), and only the mean age of ATLL onset is shifting toward older age groups, with a median age of 68 years. Stability of the number of new ATLL cases and the number of young adults seroconverting is consistent with a more important role of horizontal transmission of HTLV-1 than expected in Japan. Should they be confirmed in the decades to come, these observations indicate that even if the transmission routes of HTLV-1a-Jpn are shifting, the incidence of ATLL is stable, suggesting that virus-intrinsic properties might contribute significantly to the high incidence of ATLL in Japan.

### Host genetics

#### Indirect evidence of genetic susceptibility to HTLV-1 infection and associated diseases

Demonstrating genetic susceptibilities to HTLV-1 diseases is challenging, primarily because HTLV-1 infection itself is not homogeneously prevalent among the distinct groups from different origins included in the studies. Moreover, intra-familial transmissions inside isolated populations can bias the analysis of host genetics contributions. Also, other factors potentially influencing susceptibility to infection and to pathological manifestations, such as environmental factors or socio-economic contexts, are not systematically normalized. Studies in Japan highlighted an influence of a family history of malignancies in ATLL patients, as well as the origin of patients from endemic areas (44), consistent with the notion of genetic predisposition to ATLL, but also consistent with genetic predisposition to HTLV-1 infection itself. In Australia, it appears that most cases of ATLL are reported in non-Indigenous individuals (115), suggesting that the genetic background could significantly contribute to disease risk. However, in the absence of information on the genotype infecting these individuals, it is difficult to establish whether all these cases are associated with infection by HTLV-1c, a point that would be required to accurately disentangle the contribution of the viral genotype and the host genetic background.

More recent studies have attempted to explore the influence of host genetics on susceptibility to HTLV-1 infection or diseases in controlled cohorts of individuals, closely related either from a geographical or from a socio-economic standpoint. In the United States, for instance, a study reported that African-Americans represented the predominant ethnicity both in terms of HTLV-1 infection rates and in terms of ATLL incidence (116). Clinical and molecular characteristics of infection and disease might also vary according to the genetic background of infected individuals. For instance, a higher PVL has been observed in asymptomatic carriers genetically related to HAM/TSP patients, compared to non-related carriers (117), suggesting a significant influence of the host genetic background on the establishment of the equilibrium between viral replication and immune responses, and possibly on subsequent pathological manifestations.

An interesting case is the analysis of the somatic mutational landscape in ATLL patients from Japan or North America. The comparison of frequent somatic alterations in ATLL patients revealed that Japanese patients exhibited frequent alterations in NF-κB and T-cell signaling pathways, while North American patients exhibited frequent alterations in epigenetic and histone-modifying genes, leading to a worse prognosis (118). However, this study did not consider the viral strain circulating in these populations, and these molecular disparities could be related, at least in part, to different properties of HTLV-1a-TC and 1a-Jpn strains. Nonetheless, host genetics also undeniably contributes to these observations, as large-scale studies on ATLL patients in Okinawa, Japan, where HTLV-1a-TC is dominant, indicate that patients exhibit distinct somatic mutation susceptibilities compared to North American patients (119). In addition, patients from Okinawa and from Japan mainland, where HTLV-1a-Jpn is predominant, show very similar alteration profiles, suggesting that the viral genotype itself cannot explain all the geographical disparities in this case.

#### Host genetic factors that contribute to the geographical disparities in HTLV-1 infection outcome and interaction with the viral genotype

As mother-to-child transmission is purportedly one of the major risk factors for ATLL development, it is difficult to disentangle the genetic risk of infection by the oral route and a genetic susceptibility to ATLL (120, 121). Studies based on siblings could be informative on that matter; however, siblings may not have had the same duration of breastfeeding, or the PVL of their mother may have evolved over time and have differed at the time of birth. Nonetheless, some specific loci have been associated with an increased risk of HTLV-1 transmission to newborns (120). In addition, concordance between mother and newborn genotypes at the Human Leukocyte Antigen (HLA) loci represents an important risk for ATLL development, possibly by making infants more susceptible to vertical transmission and by reducing viral-specific immune responses in the infant (122).

In addition, several studies have focused on the association between HLA haplotype and the development of HAM/TSP. For instance, HLA-DRB1*0101 was shown to be associated with an increased susceptibility to HAM/TSP in Japanese cohorts (123–125). This correlation was hypothesized to be the result of a facilitated recognition of HTLV-1 antigens on class II MHC by T cells, leading to a stronger immune response and an increased anti-HTLV-1 envelope protein (gp21) antibody titer (126–128). Conversely, some HLA alleles, such as HLA-A*02, are associated with protection against HAM/TSP, through the reduction of PVL in carriers (108). Intriguingly, these alleles are associated with a weaker recognition of HTLV-1 antigens by the host immune system, emphasizing the intricate interplay between host genetics, immune response to HTLV-1, and control of viral replication. While the protective effect of HLA-A*02 has been recently confirmed in populations from South America and the Caribbean (129, 130), new HLA alleles associated with susceptibility or resistance to HTLV-1 infection or diseases are currently being identified in under-studied populations (129, 131, 132). Other genetic polymorphisms have been associated with susceptibility to HTLV-1 infection and diseases, such as polymorphism in IL-10, TNF-α, or cytokine genes (133, 134). Of note, the protective effect of some alleles, such as HLA-A*02, is not observable in all cohorts reported to date (132), especially in populations that are exposed to different viral genotypes, suggesting that the viral genotype and the host genetic background could interact in a yet-to-be-documented way.

As discussed above, experimental HTLV-1 infections in animal models could help untangle the role of virus-intrinsic *versus* host-intrinsic factors in HTLV-1 pathogenesis, since they could allow the normalization of host genetic backgrounds and transmission routes to focus on HTLV-1 genotypes. On the other hand, studying the diversity of infection outcomes in natural hosts, including non-human primates, could also be informative to document the species-specific host/virus interaction patterns. HTLV-1 indeed shares an evolutionary origin with Simian T-cell lymphotropic virus type 1 (STLV-1). Interestingly, while genotypes b, d, e, f, and g are found both in humans and non-human primates, genotypes a and c have only been found in humans so far (for a review, see reference 60). In addition, a group of STLV-1 has been described in Asian non-human primates only, in particular in macaques, one of the main natural hosts of STLV-1. *In silico* analyses have revealed that these strains lack one or several accessory proteins (79, 135), indicating a specific evolutionary trajectory. How these virus-intrinsic factors might interact with host-intrinsic factors such as restriction factors (136) and impact the establishment, persistence, and transmission of infection, including zoonotic transmission to humans, as well as pathogenesis, remains a matter of debate. Of note, in some simian hosts, such as baboons, PTLV-associated diseases only occur in a fraction of animals after a long period of incubation (137). The chronic nature of STLV-1 infection and the long latency period before disease development, combined with the high cost of housing primates and the ethical regulations on animal research, thus limit the number of studies conducted in naturally infected non-human primates.

## CONCLUSION

As discussed in this review, the viral genotype emerges as a parameter of importance when aiming at disentangling the factors contributing to the disparities in disease outcomes among different populations. However, there is still a significant lack of functional data on non-HTLV-1a genotypes, in particular on African genotypes and on the HTLV-1c genotype that circulates in Australia, where clinical manifestations differ notably from those in other endemic areas. In addition, despite the possible contribution of the viral genotype, most studies still neglect this parameter when comparing cohorts, focusing instead on virus-extrinsic factors such as the age at infection, the transmission routes, and the host genetics. While these factors have been linked to disease outcomes, the evidence discussed in this review suggests that they may not be sufficient to explain the observed patterns and that they may in fact interact with the viral genotype. Developing tools for animal models could aid in disentangling these factors, but models remain challenging in the HTLV-1 field.

Thus, the geographic discrepancies in HTLV-1-associated disease risks may be better understood when also considering the predominant viral strain circulating in each region.

In addition to the factors discussed in this review, additional environmental factors could affect the outcomes of HTLV-1 infection. Smoking appears as a risk factor for the development of ATLL, as demonstrated in a Japanese cohort (138). Co-infections, in particular with parasites such as *Strongyloides stercoralis*, might also influence HTLV-1 pathophysiology. More specifically, immune dysfunctions due to HTLV-1 infection are thought to promote parasitic hyperinfection and dissemination, although the exact mechanism is still a matter of debate (for a recent review, see reference 139), and in turn, *Strongyloides* co-infection may increase the severity of HTLV-1-associated diseases. Gut microbiota composition has also recently emerged as a possible environmental factor influencing the development of ATLL. Indeed, Chiba et al. reported a higher abundance of several bacterial genera, including *Klebsiella* in Japanese ATLL patients compared to asymptomatic carriers, which was correlated with an enrichment of the succinic semialdehyde synthesis pathway that could be involved in the promotion of ATLL cell expansion through the induction of oxidative stress (140). While this opens interesting questions in the field of HTLV-1 pathophysiology, additional investigations are required to confirm this causal relationship between the gut microbiome and ATLL development. While the relevance of these findings is undisputable, again, these studies have not considered the viral genotype, and interactions between these environmental factors and genetic factors of the virus are very plausible, calling for further investigations.

Finally, the rise of horizontal transmission in recent years has facilitated the spread of HTLV-1 beyond previously isolated populations. Unlike vertical transmission, which tends to restrict the virus circulation to specific familial clusters, horizontal transmission is expanding HTLV-1 infections across broader populations, making disease patterns more heterogeneous. This tendency underscores the need for a greater focus on understudied HTLV-1 genotypes to better understand the full spectrum of HTLV-1 epidemiological, virological, and immunological features, as well as HTLV-1-related diseases.
